# The stem and roots of Wilms' tumours

**DOI:** 10.1002/emmm.201202173

**Published:** 2012-12-13

**Authors:** Peter Hohenstein

**Affiliations:** 1 The Roslin Institute, University of EdinburghEaster Bush Campus, Midlothian, UK; 2 MRC Human Genetics Unit, MRC Institute of Genetics and Molecular Medicine, University of Edinburgh, Western General HospitalCrewe Road, Edinburgh, UK

**Keywords:** cancer initiating cell, cancer stem cell, childhood cancer, kidney development, Wilms' tumour

See related article in EMBO Molecular Medicine http://dx.doi.org/10.1002/emmm.201201516

The cancer stem cell (CSC) concept goes back a long way. Already in the 19th century the ‘embryonal rest’ theory proposed that cancers originate from cells that resemble those from an early embryo (for a highly recomendable review and historic overview of the CSC field; see Nguyen et al, 2012). The CSC model that eventually emerged from this has several attractive aspects for the description and treatment of cancer. By postulating that tumours harbour a small subset of cancer stem cells that give rise, through differentiation and proliferation, to the bulk of the tumour, as well as new CSC through self-renewal, the heterogeneity of tumours can be explained. Through parallels with the normal development of the corresponding normal cell types the CSC model can help describe the earliest steps in the tumourigenic process before a fully malignant tumour is formed. Finally it predicts that the CSC should be the preferred targets of therapy, but also that due to their stem cell (-like) state they are often less responsive to the usual therapies. Much early work on CSC has been done in haematopoietic malignancies, maybe not surprisingly as large parts of their developmental cascade happens and can be followed postnatally, followed by a spur in the identification of CSC from adult solid tumours in the last 10 years. The CSC field, however, started with embryonal solid tumours (teratocarcinomas; Nguyen et al, 2012). A paper in this issue of *EMBO Molecular Medicine* (Pode-Shakked et al, 2013) takes the CSC model back to its embryonic roots by identifying the Wilms' tumour CSC.

Wilms' tumours (WT) have fascinated pathologists and developmental biologists alike for a long time. Found in the kidneys of children usually before the age of five, they show structures normally found in developing, embryonic kidneys. Moreover, a subset of WT shows the ectopic development of other mesodermal tissues, like muscle, bone, cartilage and fat. As already noted by Max Wilms in 1899, this clearly identifies these tumours as a developmental problem as much as a tumourigenic one. The tumours are believed to result from a disturbance in the mesenchymal-epithelial transition at the onset of nephron formation and several genome-wide expression and epigenetic studies have confirmed the similarity between cells found in WT and cells at these earliest stages of nephron development. This suggests a role for transformed embryonic kidney progenitor cells and CSC in the origin of these tumours (reviewed in Pode-Shakked & Dekel, 2011). As many details about kidney development are known (Costantini & Kopan, 2010), WT are an excellent model to study the link between normal development and tumourigenesis through the role of CSC. Yet, the identity of a WT CSC remained elusive, partially due to difficulties in propagating WT samples in xenografts (Xn).

»… Pode-Shakked et al. now identify NCAM^+^ALDH1^+^ cells as WT CSC…«

Through an optimized Xn protocol, Pode-Shakked et al. now identify NCAM^+^ALDH1^+^ cells as WT CSC (Fig 1). Whereas >10,000 unsorted WT cells need to be injected into immune-compromised mice for Xn propagation, only 500 NCAM^+^ or 200 NCAM^+^ALDH1^+^ are required for this. NCAM^+^ cells show enrichment in early renal progenitor markers, stem cell factors and known poor prognosis factors. Moreover, Xn samples from NCAM^+^ cells can recapitulate the complex histology found in WT, including NCAM^−^ descendent cells. This capability is even maintained after serial transplantations confirming the self-renewal capacity of the cells. Together with other lines of evidence provided, this clearly identifies the NCAM^+^ALDH1^+^ cells as WT CSC. But most importantly, the authors show that targeting the NCAM^+^ population with cytotoxic drug-conjugated anti-NCAM antibodies effectively eradicates WT Xn samples. Not only does this indirectly further confirm the CSC state of the NCAM^+^ cells, it provides a potential therapeutic approach based on these findings. Despite the greatly improved survival of children with WT, patients that do relapse respond much worse to subsequent therapy and increasingly later onset malignancies are found in former WT patients decades after treatment, likely to be secondary effects of the original therapy. The demonstration of successfully targeted NCAM^+^ WT CSC has therefore important clinical implications.

**Figure 1:**
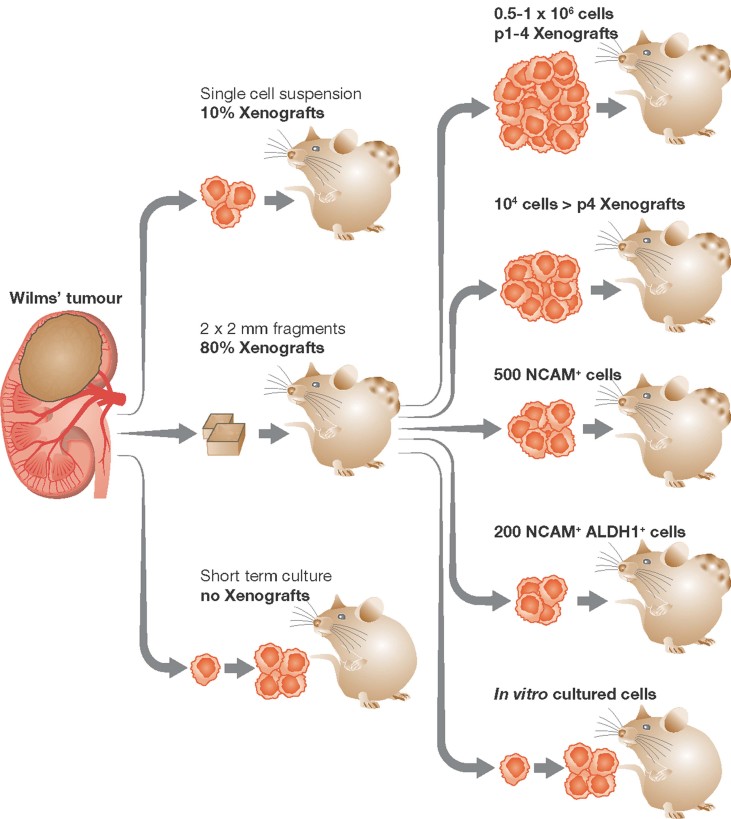
The use of small Wilms' tumour fragments in xenograft experiments greatly improves the efficiency with which these grafts are formed Once a xenograft line is established, the efficiency of propagation further increases per passage and through FACS sorting for NCAM^+^ALDH1^+^ cells.

A question that remains open is what the normal counterpart of the WT CSC is, and if these normal cells are the cells of origin of the tumours. Extensive work in mouse models has revealed that in some cancers the cell type of origin, the cell where the initiating mutation has taken place, is directly transformed into the CSC, whereas in other types of cancer the CSC is a transformed descendent of the cell of origin (Visvader, 2011). The identification of NCAM^+^ WT CSC provides clues about, but does not identify the cell of origin of these tumours. NCAM is expressed in the cap-mesenchyme cells that give rise to the complete nephron, but not much is known about the NCAM^+^ cells found there. Lineage tracing studies in mice have shown that Six2^+^ cells in the cap are the progenitors of the complete nephron (Kobayashi et al, 2008), and the WT CSC were found to be enriched for SIX2 expression. This makes the SIX2^+^ cells a good candidate for the normal, non-transformed counterpart of the WT CSC, but this has not been formally proven. As argued by the authors, the expression of ALDH1 to further define the WT CSC is likely related to the stem cell character instead of an indication of the origin of the tumour.

»A question that remains open is what the normal counterpart of the WT CSC is, and if these normal cells are the cells of origin of the tumours.«

Tied to this is the question whether the NCAM^+^ALDH1^+^ are the CSC of all WT cases. WT have often been classified in two classes (Huff, 2011). The smaller subset is characterized by loss of the *WT1* tumour suppressor gene often together with oncogenic mutations in *CTNNB1*, the gene encoding β-catenin, and these tumours tend to be found associated with intralobar nephrogenic rests. The majority of WT is wild type for *WT1* and *CTNNB1* and are found with perilobar nephrogenic rests; the initiating mutation in these tumours is not clear. Clinically these classes appear different entities altogether; the *WT1*-mutant subset of tumours shows ectopic muscle development and has the more favourable prognosis, whereas the *WT1* wild type subset is developmentally restricted to renal lineages (though clearly disturbed in their development) and has the worse prognosis.

A recent analysis of hundreds of WT samples confirmed these classes and extended them to potentially five different groups of WT (Gadd et al, 2012). This extensive dataset suggested a model where different WT classes originate from different developmental stages in the embryonic kidney. How does this fit with a CSC model? It seems logical to assume that tumour classes with different histological appearances would suggest CSC and/or cells of origin with different developmental potential. However, a direct effect of the tumour initiating mutations (and even subsequent mutations and the stage they occur) on the differentiation potential of CSC (and therefore the behaviour of the tumours) cannot be excluded. All WT CSC described by Pode-Shakked et al were derived from *WT1*-wild type tumours. More analyses will be needed to determine if the *WT1*-mutant tumours also harbour NCAM^+^ALDH1^+^ CSC. Furthermore, the genetic aberrations that drive the *WT1*-wild type tumours need to be clarified before their influence on the NCAM^+^ALDH1^+^ CSC can be taken into account.

Eventually, lineage tracing studies in mice following the fate of different cells in different mutant backgrounds will be needed to really understand the earliest steps in WT development. This will likely have important fundamental implications for our understanding of kidney development and tissue commitment in general. More important for the patients, at least in the shorter term, is that the study by Pode-Shakked et al demonstrates a new approach for the treatment of the largest and clinically most difficult class of Wilms' tumours. The identification of the NCAM^+^ALDH1^+^ WT CSC is an essential step towards a further increase in (long-term) survival of the patients.

»More important for the patients, at least in the shorter term, is that the study by Pode-Shakked et al demonstrates a new approach for the treatment of the largest and clinically most difficult class of Wilms' tumours.«

## References

[b1] Costantini F, Kopan R (2010). Patterning a complex organ: branching morphogenesis and nephron segmentation in kidney development. Dev Cell.

[b2] Gadd S, Huff V, Huang CC, Ruteshouser EC, Dome JS, Grundy PE, Breslow N, Jennings L, Green DM, Beckwith JB (2012). Clinically relevant subsets identified by gene expression patterns support a revised ontogenic model of wilms tumor: A Children's Oncology Group Study. Neoplasia.

[b3] Huff V (2011). Wilms' tumours: about tumour suppressor genes, an oncogene and a chameleon gene. Nat Rev Cancer.

[b4] Kobayashi A, Valerius MT, Mugford JW, Carroll TJ, Self M, Oliver G, McMahon AP (2008). Six2 defines and regulates a multipotent self-renewing nephron progenitor population throughout mammalian kidney development. Cell Stem Cell.

[b5] Nguyen LV, Vanner R, Dirks P, Eaves CJ (2012). Cancer stem cells: an evolving concept. Nat Rev Cancer.

[b6] Pode-Shakked N, Dekel B (2011). Wilms tumor – a renal stem cell malignancy. Pediatr Nephrol.

[b7] Pode-Shakked N, Shukrun R, Danieli MM, Tsvetkov P, Bahar S, Pri-Chen S, Metsuyanim S, Goldstein R, Rom-Gross E, Mor Y (2013). The isolation and characterization of renal cancer initiating cells from human Wilms' tumor xenografts unveils new therapeutic targets. EMBO Mol Med.

[b8] Visvader JE (2011). Cells of origin in cancer. Nature.

